# Effects of plant growth regulators and sucrose on proliferation and quality of embryogenic tissue in *Picea pungens*

**DOI:** 10.1038/s41598-023-39389-8

**Published:** 2023-08-14

**Authors:** Fang Gao, Xi Cao, Caiyun Qin, Shigang Chen, Jufeng Cai, Changbin Sun, Lisheng Kong, Jing Tao

**Affiliations:** 1 Jilin Provincial Academy of Forestry Sciences, 3528 Linhe St., Changchun, 130033 Jilin China; 2 College of Horticulture of Jilin Agricultural University, 2888 Xincheng St., Changchun, 130118 Jilin China; 3 Changchun Academy of Forestry, 5840 Jingyue St., Changchun, 130117 Jilin China; 4 Centre for Forest Biology, Department of Biology, University of Victoria, Victoria, BC V8W 3N5 Canada

**Keywords:** Biotechnology, Developmental biology, Plant sciences

## Abstract

Embryogenic tissue (ET) is important for genetic modification and plant re-generation. The proliferation ability and vigor of ET are crucial for plant propagation via somatic embryogenesis. In this study, ET was induced from mature zygotic embryos in blue spruce (*Picea pungens* Engelm.). There were significant differences in ET induction between two provenances, i.e. 78.8 ± 12.5% and 62.50 ± 12.8% respectively. Effects of 2,4-Dichlorophenoxy acetic acid (2,4-D), 6-Benzyl amino-purine (6-BA) and/or sucrose on ET proliferation and somatic embryo (SE) maturation were further investigated with four cell lines. The highest ET proliferation rate reached 1473.7 ± 556.0% biweekly. Concentrations of 2,4-D or 6-BA applied at tissue proliferation stage impacted SE maturation among the cell lines, whereas sucrose showed less effects. The highest rate, 408 ± 230 mature SEs/g FW, was achieved in SE maturation cultures. This research demonstrated that the culture conditions, i.e. the specific concentrations of 2,4-D and BA, at ET proliferation stage affected not only ET growth, but also the quality of ET for SE maturation. This study revealed the necessity and benefit in developing both the general and the genotype-specific protocols for efficient production of mature SEs, or somatic plants in blue spruce.

## Introduction

The needles of blue spruce (*Picea pungens* Engelm.) are silver-blue in color throughout the year, which is of high ornamental value for landscaping^1^. At present, blue spruce is generally regenerated via seed germination in China^2,3^. Due to the high price of seeds and seedlings, the number of introduced elite varieties is limited^4^. The needles of the progeny could be different in color, shape, structure and growth speed, and the quality of seedlings is thus limited. Technology of somatic embryogenesis can be used to produce a large number of somatic plants with consistent genetic traits in a relatively-short time period^5^, which is thus powerful in large-scale propagation of elite genotypes in blue spruce.

At present, many tree species could be propagated via somatic embryogenesis. The first success of somatic embryogenesis in coniferous species can be dated back to 1985, when Hakman et al.^6^ used immature zygotic embryos of Norway spruce (*Picea abies*) to induce embryogenic tissue (ET) and then obtained mature somatic embryos (SEs). Several articles have been published since 2000 on optimization of somatic embryogenesis in coniferous species, including: *Pinus radiata*^7^, *Picea abies*^8^, *Picea glauca*^9^, *Larix hybrids*^10^, Korean pine^11–14^, *Abies nebrodensis*^15^, *Abies nordmanniana*^16^, *Abies alba*^17^ and blue spruce^1^. The proliferation of ET together with SE maturation are key steps for large-scale propagation and longterm preservation of coniferous germplasm^15^. Nielsen et al.^16^ reported strong clonal effects in most of the traits during the propagation process via SEs in *Abies nordmanniana*^16^. The serious cell line dependence between ET proliferation and SE maturation was also reported in *Abies alba*^17^. Such kind of research was not reported previously in blue spruce.

There have been few research reports on the initiation of somatic embryogenesis in blue spruce. Afele et al.^18^ used mature blue spruce embryos, as explants, for ET induction. The highest ET induction rate was 16%, and the number of somatic embryos (SEs) produced was only 9.7 SEs/g FW. Sun et al.^4^ further optimized the ET induction system. They achieved the induction rate up to 45.5%. However, in the following stages, there were many abnormal embryos resulting in poor SE germination ability. Hazubska-Przyby et al.^19^ induced ET from zygotic embryo with the highest induction rate of 23.75%, whereas no mature SEs was obtained. Tao et al.^1^, at the first time, developed a full technical system of somatic embryogenesis in blue spruce, including the cultures of ET induction and proliferation, SE maturation, SE germination and conversion, as well as the hardness of somatic plants. However, the technical system of somatic embryogenesis in blue spruce needs further optimization to solve the problems, such as the low rates of ET induction and proliferation, as well as the poor SE maturation ability. All of these factors limited the large-scale propagation process via somatic embryogenesis in blue spruce. The essence of ET proliferation is the cell division of proembryogenic mass. Factors such as 2,4-Dichlorophenoxy acetic acid (2,4-D), 6-Benzyl amino-purine (6-BA) and sucrose showed great influences on ET proliferation^20^, and so do the culture conditions at ET proliferation stage. However, there were few studies on their effects on SE maturation thereafter. There are various factors influencing SE maturation^21,22^. It is essential to study the effect of culture conditions at ET proliferation stage on SE maturation in order to optimize the entire culture system for mass propagation. In this study, mature zygotic embryos of blue spruce from two provenances were used as explants to induce ET, and the induced ETs were used as experimental materials to explore the effects of 2,4-D, 6-BA and/or sucrose on ET proliferation rates and the ET quality on SE maturation in order to optimize the SE system for efficient production of elite somatic plants in blue spruce.

## Materials and methods

### ET induction

Mature blue spruce seeds from two provenances were labeled as F1 and F2, and stored in a -40 ℃ freezer before use. The seeds were purchased from Carson Forest (provenance F1), NM, USA in 2010, or San Isabel (provenance F2), CO, USA in 2008. The seeds were of different genotypes, which were produced by various mother trees via open-pollination. Seeds for the test were rinsed with running tap water for 18 h, and then placed on an ultra-clean workbench, sterilized with 75% alcohol for 30 s. The seeds were then rinsed with sterile distilled water for 3–5 times, and sterilized with NaClO solution (4% chlorine) for 15 min, before being rinsed 3–5 times with sterile water. The seed coat and megagametophytes were removed using a scalpel. The embryos were taken out, being used as explants. The dissected embryos were placed on the surface of culture medium horizontally. The ET induction medium was the modified LV (mLV) medium^23,24^ supplemented with 4.0 mg/L 2,4-D, 1.0 mg/L 6-BA, 10 g/L sucrose, 0.8 g/L acid -hydrolyzed casein (Casein Hydrolysate, CH), 0.5 g/L l-glutamine, and 4 g/L Gedrite.

### Experiments with the different concentrations of plant growth regulators

#### Effects of 2,4-D on ET proliferation

Two moths after being cultured with the proliferation medium, the induced ET was used for the experiments of ET proliferation and SE maturation. A new medium was replaced every two weeks, and four selected cell lines were used, including cell lines 2#04, 2#13, 1#16 and 2#02. Among them, cell line 1#16 was induced from F1 source, while cell lines 2#04, 2#13 and 2# 02 were induced from F2 source. The ET of transparent filamentous morphology was selected for subcultures in order to obtain as much ET as possible for subsequent tests. The mLV medium was used for ET proliferation, which was supplemented with 1.0 mg/L 6-BA and 2.0 mg/L 2,4-D. Other culture conditions were the same as the described in Section “ET induction”.

For the experiment of 2,4-D test, culture medium of mLV was used as the basal medium. It was supplemented with 0, 1.0, 2.0, 3.0, 4.0, or 6.0 mg/L 2,4-D, 1.0 mg/L 6-BA. The other culture conditions were the same as the described in 2.1. Five Petri dishes, as the minimum, were used for data collection for each treatment and each cell line. The experiment was repeated when it was necessary. The initial ET for inoculation was 0.2 g per Petri dish. After 14 days, the fresh weight (g) of ETs in each dish was weighed in order to calculate ET proliferation rate on the following formula.

$$ {\text{ET}}\;{\text{proliferation rate}}\;\left( \% \right) = \left( {{\text{fresh }}\;{\text{weight }}\;{\text{after}}\;{\text{ proliferation}} - {\text{ fresh }}\;{\text{weight}}\;{\text{ before}}\;{\text{ proliferation}}} \right) \, /{\text{ fresh }}\;{\text{weight }}\;{\text{before }}\;{\text{proliferation }}*{1}00. $$

#### Effects of 6-BA on ET proliferation

The same four cell lines were used for this experiment. In the basal mLV medium, 0, 0.5, 1.0, 2.0, or 3.0 mg/L 6-BA was added respectively with 2.0 mg/L 2,4-D. Other culture conditions were the same as the described in Section “ET induction”. Five Petri dishes were used for each treatment with each cell line.

### Experiment with different sucrose concentrations

The same cell lines were used for the experiment. In the basal medium, 5, 10, 20, or 30 g/L sucrose was added respectively with 2.0 mg/L 2,4-D and 2.0 mg/L 6-BA. Other culture conditions were the same as the Section “ET induction”. Five Petri dishes were used for each treatment with each cell line.

### Maturation

In proliferation cultures, ET of the same four cell lines was treated based on the described in the Sections “Experiments with the different concentrations of plant growth regulators” and “Experiment with different sucrose concentrations”. The ET after the treatments for 7–10 days in proliferation cultures was used for SE maturation in order to determine ET quality. Approximate 80 mg ET was added into 4 ml liquid medium, which was the proliferation medium without plant growth regulators and the solidifying agent. The tissue was mixed well with the culture medium. After the mixture was filtered, the filter paper with ET on it was placed on SE maturation medium in a Petri dish of 10 cm in diameter. For SE differentiation and maturation, the basal medium (mLV) was supplemented with 13.22 mg/L abscisic acid (ABA), 30 g/L sucrose, 6 g/L gelrite, 0.8 g/L CH, 0.5 g/L l-glutamine, and 2 g/L activated charcoal (AC). Five Petri dishes were used for each treatment with each cell line. The culture was kept in the dark at 23 ± 2 °C, and the number of SEs was counted after a 60-d SE maturation culture. Somatic embryos with well-developed cotyledons were counted as mature SEs.

### Observation of ET morphology

Plant tissues were collected at various culture stages including the tissue from ET proliferation culture for 7–10 days (early immature SEs); tissues in SE maturation culture for 5 days (stage I), 10 days (Stage II), 30 days (stage III), 45 days (stage IV), and 60 days (stage V). The plant materials (0.1 g ET) at each stage were stained with 0.1% safranin solution for 10 min and placed on a glass slide, dripped with a drop of water, and covered with a cover slip. The ET was spread evenly by tapping lightly with the flat end of a pencil, and observed immediately and photographed with a microscope (OLYMPUS CX 31, Japan; equipped with Canon DS126271 camera, Japan).

### Statistic and data analysis

Calculations were performed using Excel 2003 (Microsoft, United States). One-way analysis of variance (ANOVA) and Duncan’s multiple comparisons tests were performed using SPSS 19 (IBM, United States). Graphs were constructed with Sigma Plot 12.0 (Systat, United States).

### Research involving plants

The authors declare that the study on plants in this research, including the collection of plant materials, complies with relevant institutional, national, and international guidelines and legislation.

## Results

### ET induction

**Figure 1:**
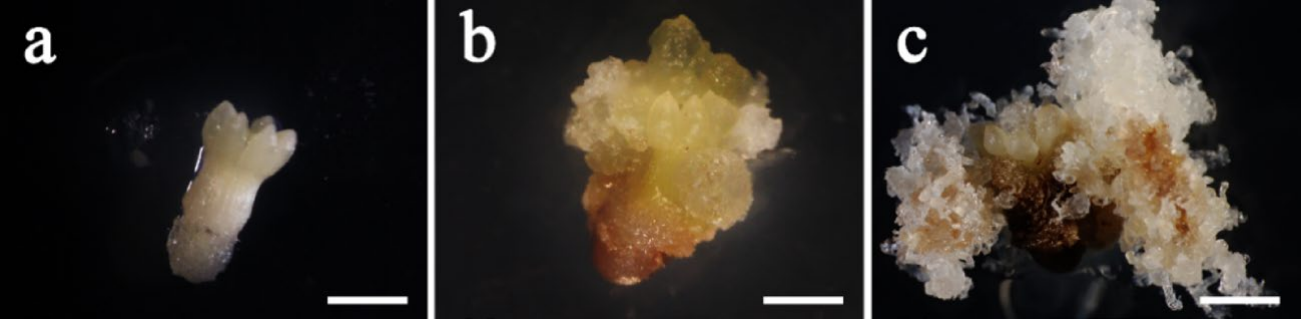
The process of ET induction in blue spruce. (**a**) a zygotic embryo on ET induction medium for 7 days; (**b**) Non-embryogenic callus in the induction culture for 60 days; (**c**) Embryogenic tissue in the induction culture for 60 days. All bars = 3 mm.

After being placed on ET induction medium for 7 days, zygotic embryos, as explants, expanded and the cotyledons enlarged (Fig. 1a). Non-embryonic callus (NEC) and ET were induced under the treatments supplied with 2,4-D of different concentrations. The surface of NEC was hard, yellow-green, and grainy (Fig. 1b), whereas ET appeared as white fluffy filaments (Fig. 1c). The ET induction rate achieved was 62.50 ± 12.8% or 78.8 ± 12.5% with provenance L1 or provenance L2 respectively.

### ET prtoliferation and SE development

**Figure 2:**
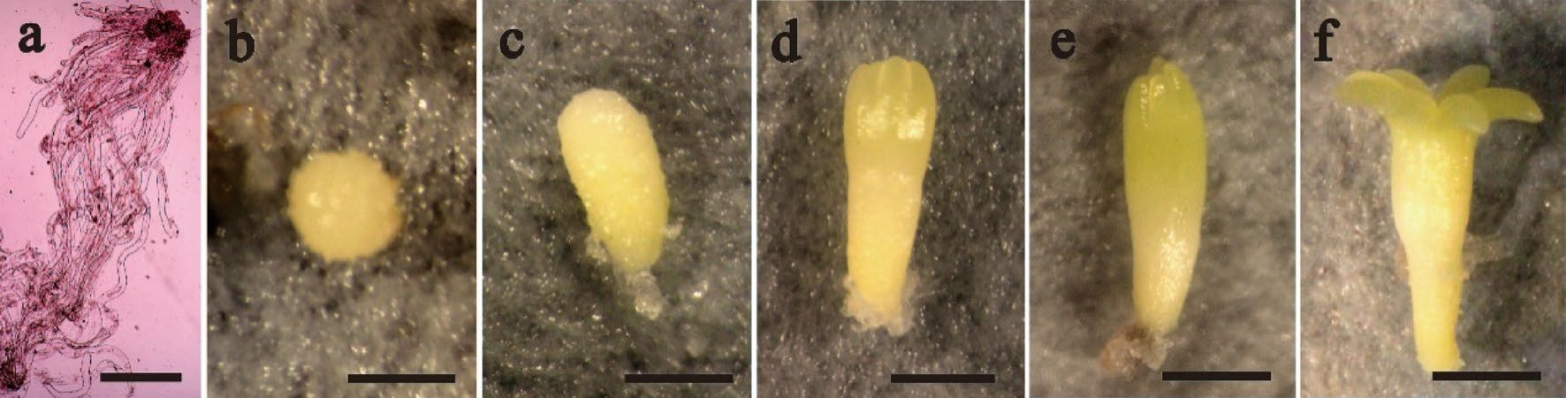
Somatic embryos at different developmental stages in blue spruce. (**a**) Early immature SEs in ET proliferation culture for 7 to10 days, bar = 50 μm; (**b**–**f**) The SE in maturation culture for 5 days (**b**), 10 days (**c**), 30 days (**d**), 45 days (**e**), and 60 days (**f**) respectively. Bars (**b**–**f**) = 2 mm.

Various stages of SE development and maturation are shown in Fig. 2. The early immature SEs existed and multiplied at ET proliferation stage (Fig. 2a) after a 7 to10-day proliferation culture and the ET was used to stimulate SE maturation. In maturation culture for 5 days, SEs reached embryo developmental stage I and the SEs were spherical (Fig. 2b). The SEs developed into the Stage II after 10 days in the mature culture, and the SE was cylindrical (Fig. 2c). After being cultured for 30 days, the SEs started to develop cotyledons at stage III (Fig. 2d). The cotyledons continued to elongate after SEs were cultured for about 45 days on maturation medium at stage IV (Fig. 2e). Somatic embryos matured fully with well-developed cotyledons at developmental stage V, after 60 days in the culture (Fig. 2f).

### Effects of 2,4-D on ET proliferation and SE maturation

**Figure 3:**
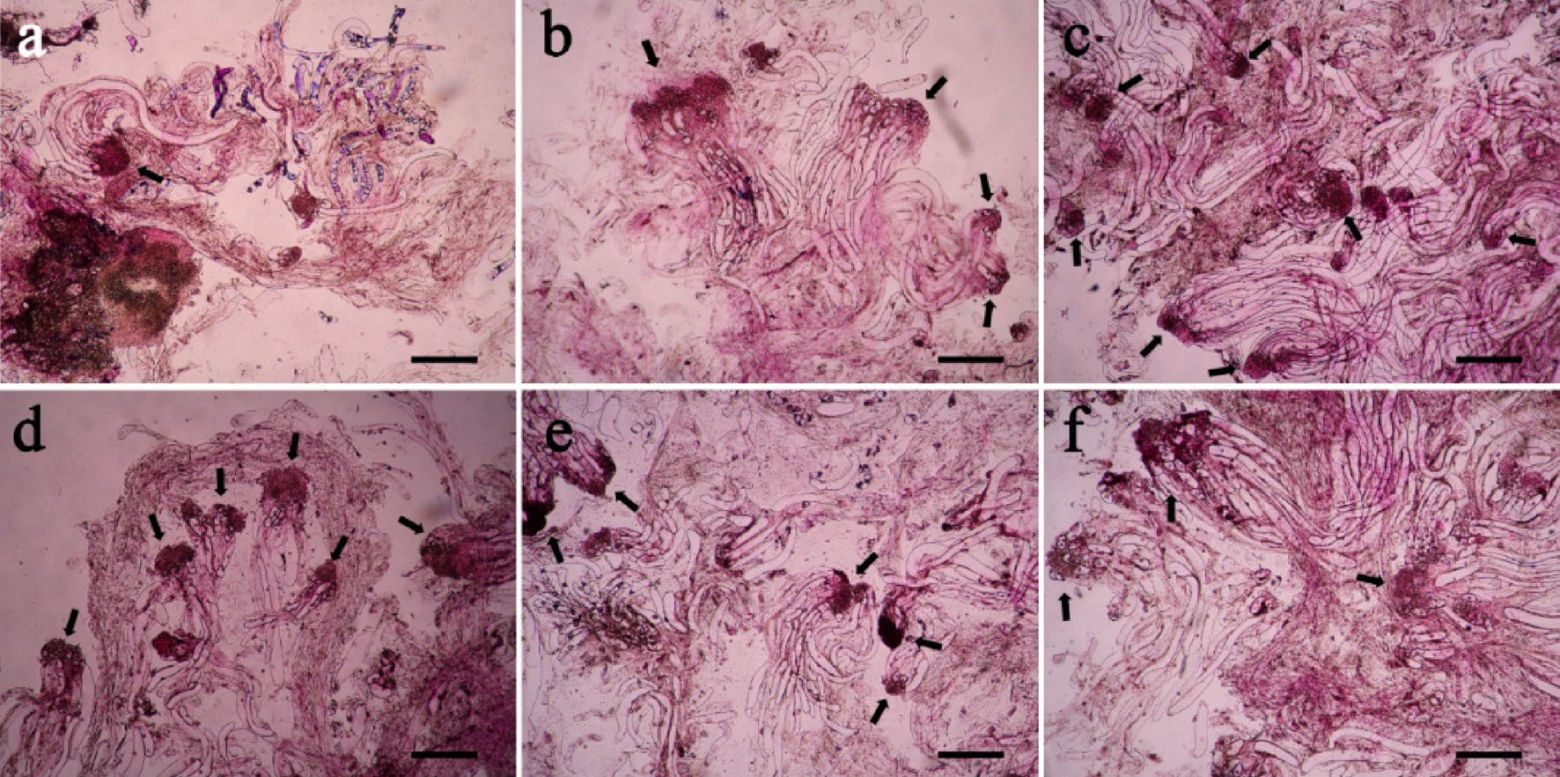
Effects of 2,4-D on ET structure during proliferation in blue spruce. (**a**) ET in the culture when 2,4-D was absent from the medium; (**b**) ET in proliferation culture supplemented with 1.0 mg/L 2,4-D; (**c**) ET in prolifera-tion culture supplemented with 2.0 mg/L 2,4-D; (**d**) ET in proliferation culture supplemented with 3.0 mg/L 2,4-D; (**e**) ET in proliferation culture supplemented with 4.0 mg/L 2,4-D; (**f**) ET in proliferation culture supplemented with 6.0 mg/L 2,4-D. Arrows point at somatic embryos. All bars = 50 µm.

When 2,4-D was not supplemented in the culture medium, few early immature SEs was observed in ET and the structure of SEs was poor (Fig. 3a), since the SEs consisted of small, loos embryo propers and less organized suspensors; When 2,4-D concentrations arranged from 1.0 to 4.0 mg/L, more SEs were estimated than the culture without 2,4-D in the medium (Fig. 3b–e); the number of early SEs in the ET started to decrease when 6.0 mg/L 2,4-D was supplemented (Fig. 3f), as compared to Fig. 3c–e. In all the six treatments, the optimum 2,4-D concentration was 3.0 mg/L (Fig. 3d), with which the highest SE number and the best SE morphological characteristics, i.e. the dense embryo proper with a well-developed suspensor, were obtained.

**Figure 4:**
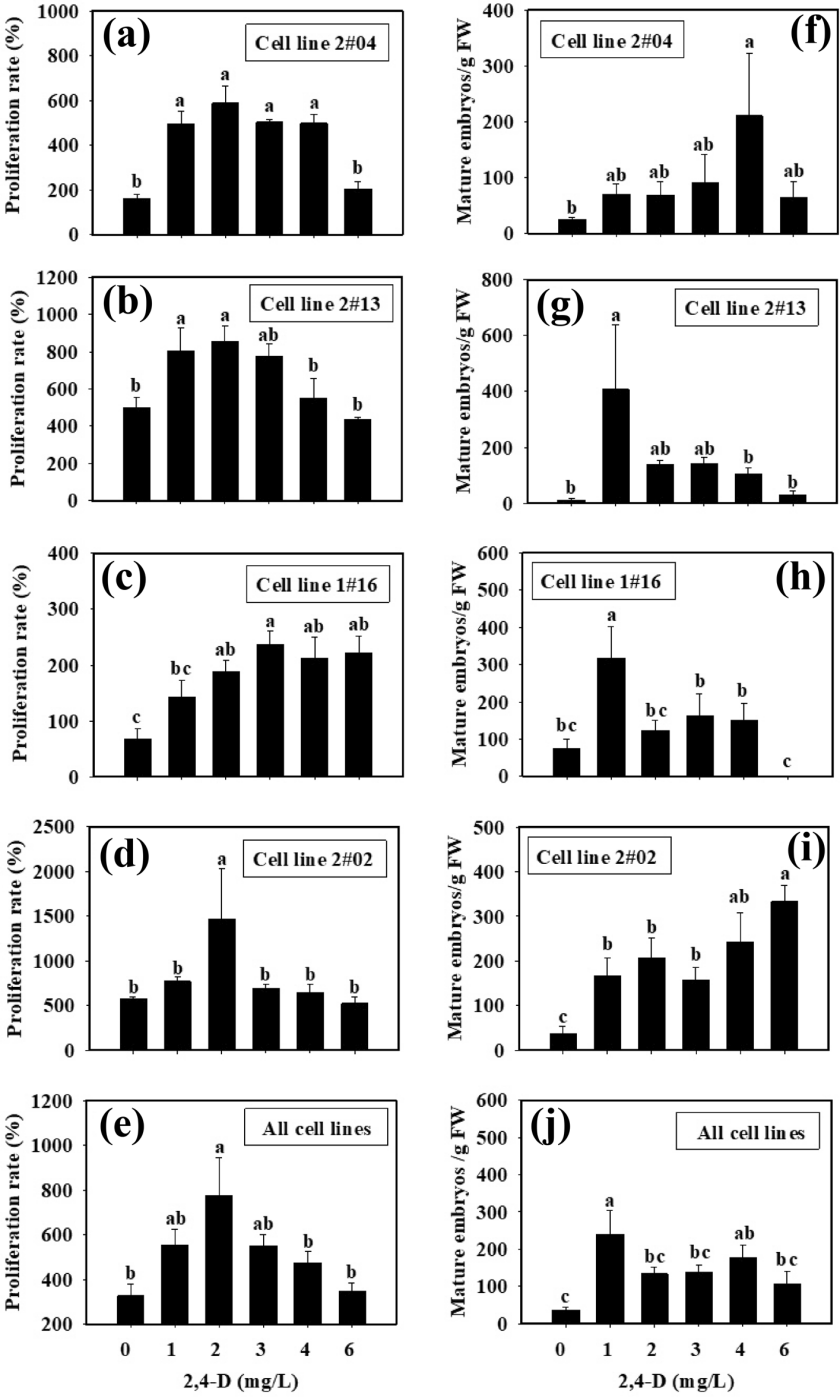
Effects of 2,4-D concentrations supplemented at tissue proliferation stage on ET proliferation and SE maturation with different cell lines in blue spruce. (**a**) Proliferation of cell line 2#04; (**b**) Proliferation of cell line 2#13; (**c**) Proliferation of cell line 1#16; and (**d**) Proliferation of cell line 2#02; (**e**) Proliferation of all cell lines; (**f**) Maturation of cell line 2#04; (**g**) Maturation of cell line 2#13; (**h**) Maturation of cell line 1#16; and (**i**) Maturation of cell line 2#02; (**j**) Maturation of all cell lines. Mean ± SE, N = 5 for individual lines or 20 for all the four lines, different lowercase letters indicate significant difference at *p* ˂ 0.05.

Significant difference existed between the cultures of the control (2,4-D was absent from the culture medium) and those containing 2,4-D of some specific concentrations (Fig. 4a–e). When 2,4-D concentrations were in the range of 0.0–2.0 mg/L, the proliferation rate increased with the increase of 2,4-D in all the cell lines (Fig. 4a–d). When 2,4-D concentrations arranged between 0.0 and 3.0 mg/L, the proliferation rate of cell line 1#16 increased with the higher 2,4-D concentrations, while the proliferation rate decreased when 2,4-D concentration exceeded 4.0 mg/L (Fig. 4c). With cell line 2#04, 4 mg/L 2,4-D seems to give high number of mature SEs. However, it was only statistically significant to the control without 2,4-D supplememnted to the culture. With all of the four cell lines, the highest proliferation rate was achieved when 2.0 mg/L 2,4-D was added into the culture medium (Fig. 4e).

In SE maturation cultures, significant difference existed between the control and the treatments of some specific concentrations of 2,4-D (Fig. 4f–g). When 2,4-D concentrations ranged between 1.0 and 4.0 mg/L (Fig. 4a), there was no significant difference in SE development and maturation with cell line 2#04 (Fig. 4f); Cell line 2#13 showed higher proliferation capacity than the others with 2,4-D concentrations between 1.0 and 2.0 mg/L (Fig. 4b), and the ET had the highest proliferative capacity at 2.0 mg/L, whereas 1.0 mg/L 2,4-D resulted in the highest number of mature SEs (408 ± 230 mature SEs/g FW) (Fig. 4g); cell line 1#16 had the highest proliferation ability when 2,4-D concentration was 3.0 mg/L (Fig. 4c), whreans at 1.0 mg/L, SE development/maturation ability was the best (318 ± 84 mature SEs/g FW) (Fig. 4h); cell line 2#02 showed the highest proliferation ability at 2,4-D concentration of 2.0 mg/L (proliferation rate reached up to 1473.7 ± 556.0%, Fig. 4d), whereas at 6.0 mg/L, the SE development/maturation ability was the best (333 ± 37 mature SEs/g FW) (Fig. 4i). With all of the four cell lines, the highest SE maturation ability was achieved when 1.0 mg/L 2,4-D was supplemented in the culture medium (Fig. 4j).

### Effects of 6-BA on ET proliferation and SE maturation

**Figure 5:**
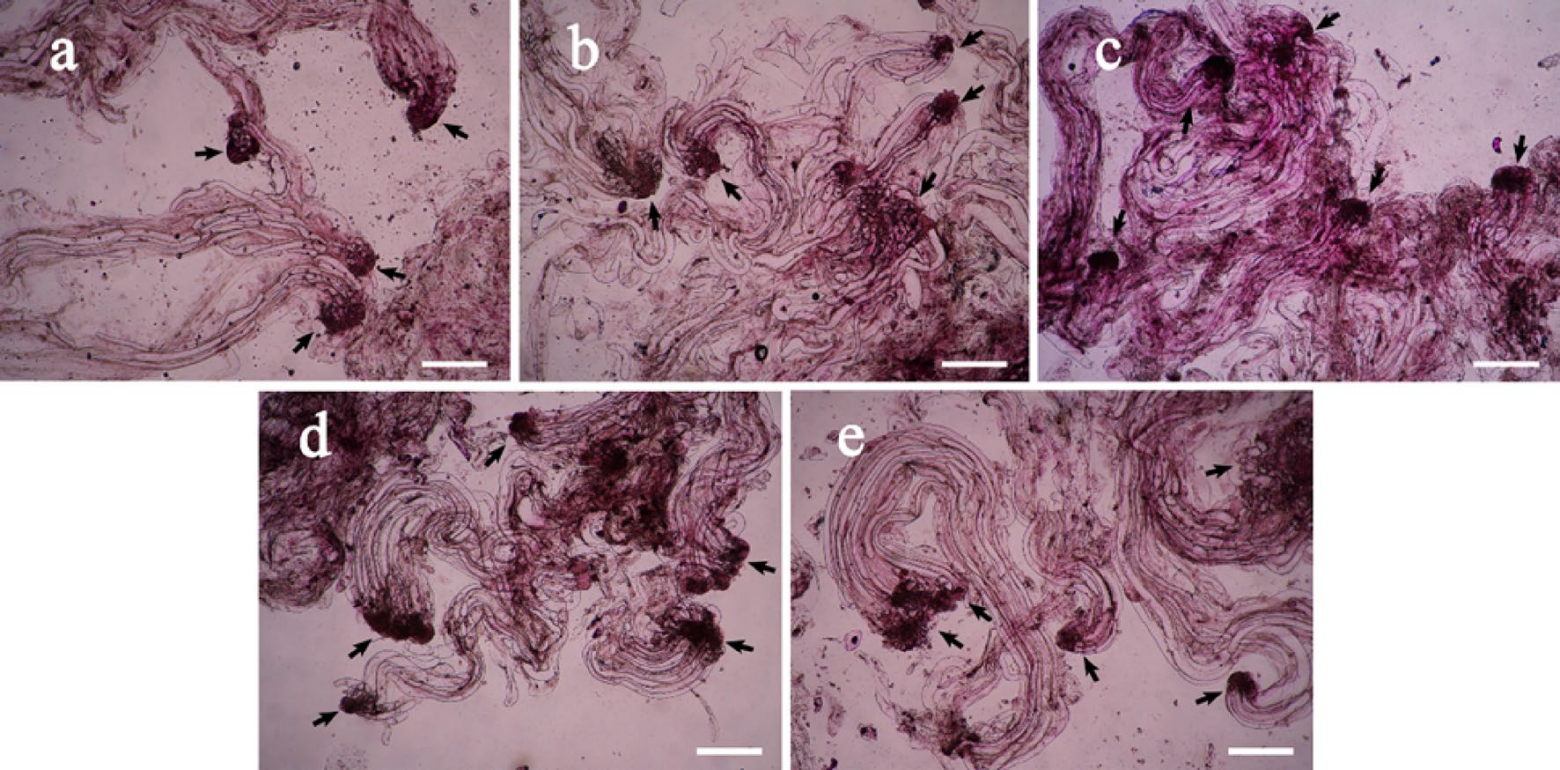
Effects of 6-BA concentrations on SE differentiation ability in blue spruce. (**a**) ET proliferation in the culture without 6-BA; (**b**–**e**) ET in the proliferation culture of 0.5 mg/L 6-BA, 1.0 mg/L 6-BA (**c**), 2.0 mg/L 6-BA (**d**), and 3.0 mg/L 6-BA (**e**) respectively. Arrows point at somatic embryos. All bars = 50 µm.

**Figure 6:**
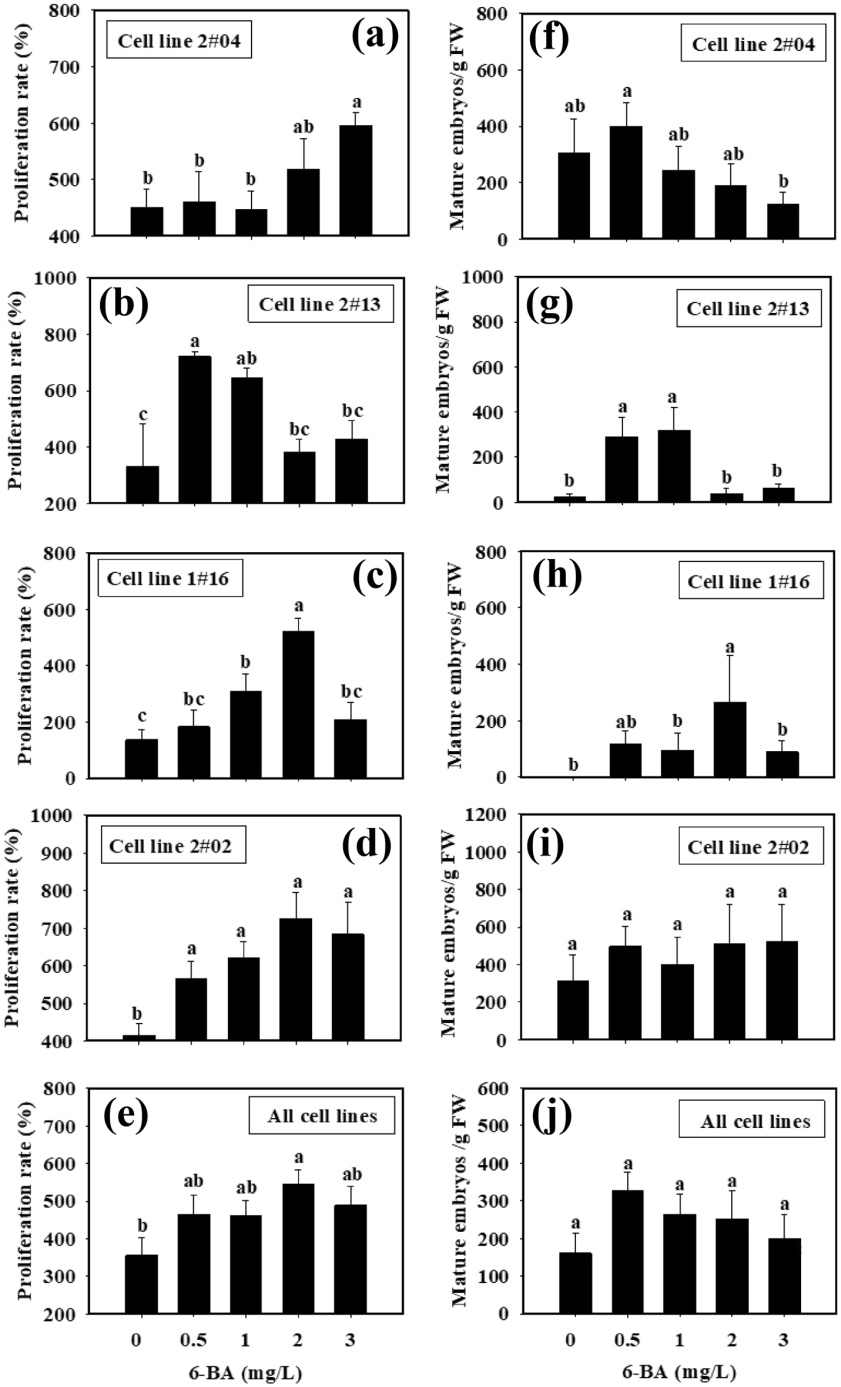
Effects of 6-BA concentrations supplemented at tissue proliferation stage on ET prolif-eration and SE maturation with different cell lines in blue spruce. Mean ± SE, N = 5 for individual lines or 20 for all the four lines. Different lowercase letters indicate significant difference at *p* ˂ 0.05.

The effect of 6-BA concentration on ET structure of the four cell lines is shown in Fig. 6. When no 6-BA was added to the medium, the number of early SEs in ET was less than that of the tissue treated with 1 mg/L 6-BA (Fig. 5a). The embryo proper was well-organized, but the SEs were tiny; When 6-BA concentrations ranged from 0.5 to 3 mg/L, the difference in the number of early SEs in ET was not obvious (Fig. 5b–e). When 2 mg/L 6-BA was supplemented in the culture, the early immature embryos looked healthy, because the size of SE was relatively larger, the cells were densely packed in the embryo proper, and the suspensors were clearly defined.

Significant difference existed between the control (6-BA was absent from the culture medium) and the treatments of some specific 6-BA concentrations (Fig. 6a–e). It was not beneficial for ET proliferation when 6-BA concentrations were too high or too low (Fig. 6). With the increase of 6-BA concentrations from the zero, the ET proliferation rate increased first and then decreased later Cell line 2#04 had a higher proliferation rate when 6-BA concentration was 2.0–3.0 mg/L (Fig. 6a). Cell line 2#13 showed the highest proliferation rate (721.0 ± 15%) when 0.5 mg/L 6-BA was supplemented (Fig. 6b); Cell line 1#16 had the highest proliferation rate (520.0 ± 49.8%) when 6-BA concentration was 2.0 mg/L (Fig. 6c), while cell line 2#02 achieved better proliferation rates when 6-BA concentrations ranged between 0.5 and 3.0 mg/L (Fig. 6d). With all of the four cell lines, the highest ET proliferation rate was achieved when 2.0 mg/L 6-BA was supplemented in the culture medium (Fig. 6e).

When 6-BA concentration was 2.0 or 3.0 mg/L, cell line 2#04 demonstrated higher proliferation rates, whereas the SE maturation ability was poor. When 0.5 mg/L 6-BA was supplemented, the ability of SE maturation was the best (400 ± 83 mature SEs/g FW) (Fig. 6f). With cell line 2#13, the ability of SE maturation was the best when 6-BA concentration was 1.0 mg/L (320 ± 101 mature SE/g FW) (Fig. 6g); For cell line 1#16, 2.0 mg/L 6-BA resulted in the highest proliferation rate, whereas no significant difference was observed in the ability of SE maturation (Fig. 6h); In cell line 2#02, no significant difference was found in SE maturation with different 6-BA concentrations applied at the proliferation stage (Fig. 6i).

### Effects of sucrose on ET proliferation and SE maturation

**Figure 7:**
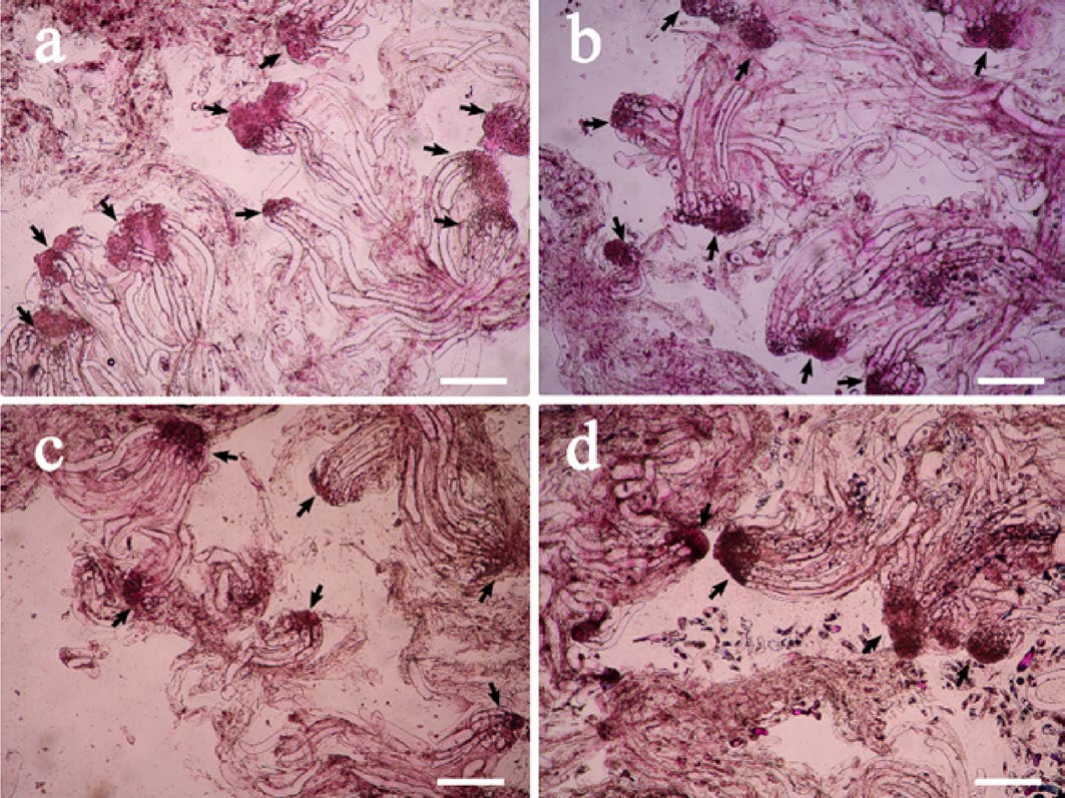
Effects of sucrose concentrations on SE formation in ET at tissue proliferation stage in blue spruce. (**a**–**d**) ET proliferation culture supplemented with 5 g/L sucrose (**a**), 10 g/L sucrose (**b**), 20 g/L sucrose (**c**), and 30 g/L sucrose, respectively. Arrows point at early immature somatic embryos. All bars = 50 µm.

The effects of different sucrose concentrations on the structure/morphology of ET in the four cell lines are shown in Fig. 7. When sucrose concentration was 5 or 10 g/L, the number of early immature SEs in the ET was higher than those of the other concentrations and the morphological characteristics of the SEs looked better (Fig. 7a,b) with more organized structures. When sucrose was increased to 20 g/L, the number of SEs in the ET reduced (Fig. 7c). When 30 g/L sucrose was supplemented, the number of SEs in ETs decreased clearly (Fig. 7d), when compared to Fig. 7b and c.

**Figure 8:**
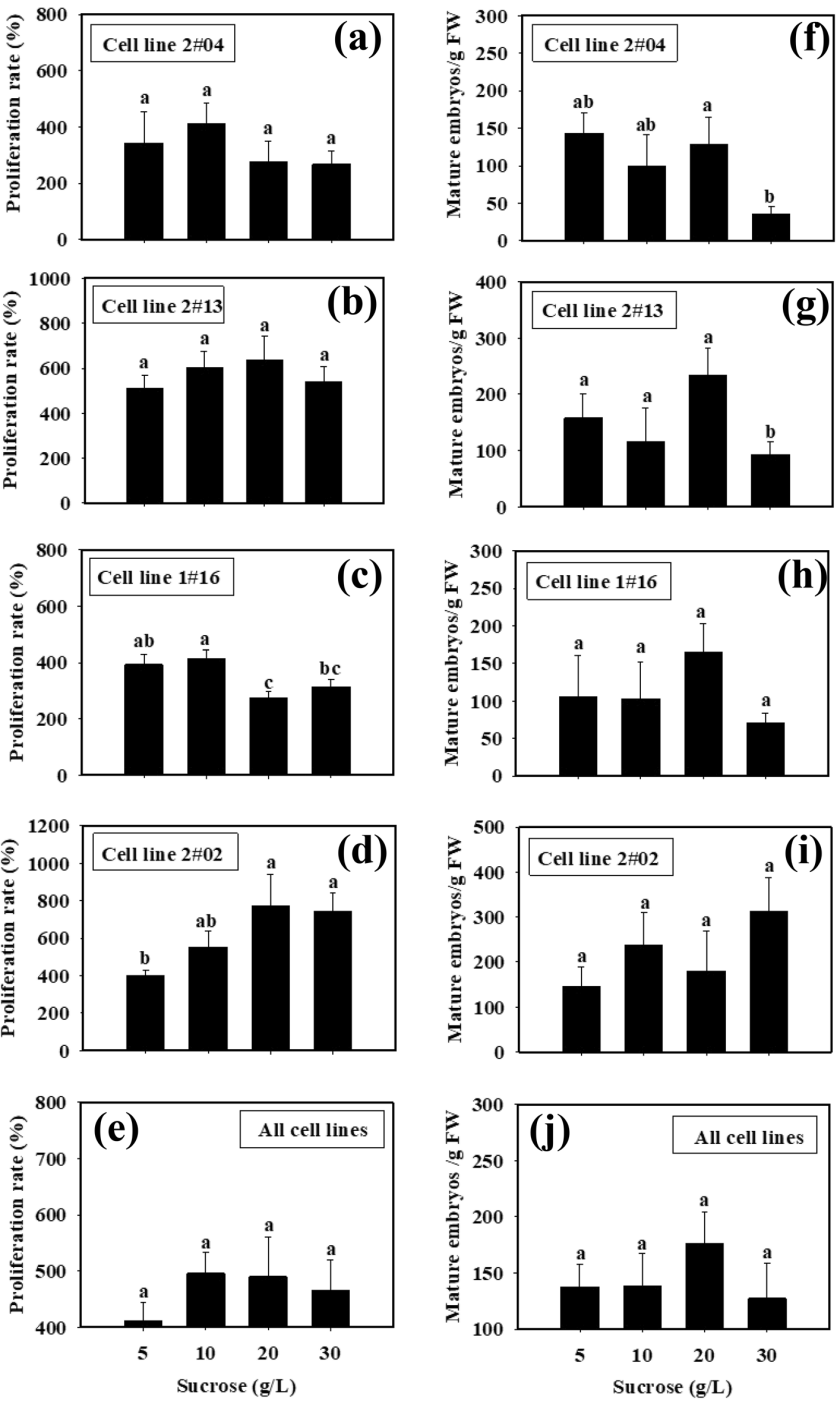
Effects of sucrose concentrations supplemented at tissue proliferation stage on ET proliferation and SE maturation with different cell lines in blue spruce. Mean ± SE, N = 5 for individual lines or 20 for all the four lines, different lowercase letters indicate significant difference at* p* ˂ 0.05.

Significant difference of ET proliferation between the treatments of some specific sucrose concentrations was found in cell lines 2#02 and 1#16. In the line 2#02, the better tissue proliferation was obtained with the higher sucrose concentrations (Fig. 8d), whereas it was opposite with cell line 1#16 (Fig. 8c). Cell line 2#04 had a higher ET proliferation rate (412.6 ± 74.0%) when 10 g/L sucrose was used (Fig. 8a), whereas it was 20 g/L sucrose for cell line 2#13 (Fig. 8b). Cell line 1#16 achieved the highest proliferation rate (413.4 ± 30.2%) when 10 g/L sucrose was supplemented (Fig. 8c). For cell line 2#02, the proliferation rates were higher with 10 to 30 g/L sucrose than 5 g/L sucrose (Fig. 8d). With all of the four cell lines, the highest ET proliferation rate was achieved when 10 or 20 mg/L sucrose was supplemented in the culture medium (Fig. 8e). Significant difference of SE maturation between the treatments of some specific sucrose concentrations (Fig. 8f-i) could be found only in cell lines 2#04 (Fig. 8f) and 2#13 (Fig. 8g). With these two lines, the higher concentrations of sucrose reduced SE maturation ability. No significant difference was found in the sucrose test with all of the four cell lines together (Fig. 8j).

## Discussion

Plant growth regulators (PGRs) play an important role in different species during the entire process of somatic embryogenesis^25–28^, such as ET induction (*Phoenix dactylifera*), ET proliferation (*Pinus sibirica* and *Larix sibirica*) and SE development/maturation (*Medicago sativa*)^24,29,30^. Therefore, it is crucial to gain insight into the potential effects of exogenously-applied PGRs on somatic embryogenesis. 2,4-D, functions as auxin, is considered to be the most critical regulator during ET induction in conifers^31,32^. In this study, when 2,4-D was absent from the culture medium, ET proliferation rates of the four cell lines were all low, and SE maturation ability, at the following stage, was also poor. Thus, it was confirmed that the supplement of 2,4-D is essential for ET proliferation. Significant differences were also found in SE maturation in certain cell lines. When 2,4-D concentration reached at 6.0 mg/L, the number of early immature SEs decreased in ET. At SE maturation stage, except for cell line 2#02, the number of mature SEs/ g FW dropped remarkably. Cell line 1#16 even lost its embryogenicity. These demonstrated the fact that if 2,4-D concentration is too high, either ET proliferation or SE maturation will be impacted negatively.

Many evidences have proved that the simultaneous use of cytokinin and auxin during ET proliferation is more effective than the single use and these two PGRs are synergistic to a certain extent, such as in *Acacia arabica*^33^. In this experiment, when 6-BA was absent from the medium, ET proliferation rate and SE maturation ability were lower than that of the 6-BA-added, in the four cell lines. With the increase of 6-BA concentrations, ET proliferation rates increased gradually. Thus, 6-BA is necessary for ET proliferation although the SE maturation ability showed no significant difference. In comparison, 2,4-D concentrations demonstrated a greater effect on SE proliferation rate than 6-BA. When 6-BA exceeded 2.0 mg/L, ET proliferation rate decreased in cell line 2#13. In cell line 1#16, when 6-BA exceeds 3.0 mg/L, both the proliferation rate and the SE maturation ability declined. All of the facts reveal that the optimum concentration of 6-BA plays an important role. Meanwhile, this study also found that, it is well given 2,4 D as well as BA affect the proliferation rate, but it is strongly cell line dependent.

For conifer embryos, sucrose concentrations in the range of 1%-3% resulted in better development^34^. For some tree species, glucose and maltose are preferable carbon sources, for example, 2% maltose was better for ET induction than 2% sucrose in *Pinus nigra*^35^. High concentrations of sucrose could cause cell plasmolysis, thereby causing dehydration and death of poorly resistant cells^36^. In this study, the responses to sucrose of various concentrations were different in the four cell lines. When sucrose was 10 g/L, ET proliferation rates were higher than other concentrations in all the four cell lines. When sucrose was in the range of 5–30 g/L, the SE maturation ability was not significantly different in either cell lines 1#16 or 2#02. When sucrose concentration increased up to 30 g/L, SE maturation ability of cell lines 2#04 and 2#13 declined. However, a certain number of early immature SEs could be observed under all the treatments of sucrose concentrations, indicating that the concentration of sucrose was not the major factor promoting early SEs. The number of early SEs in ET decreased when sucrose reached at 30 g/L, which may attribute to the high osmotic potential of the culture, resulting in poor ET proliferation.

On this study, there were significant differences in ET induction between the two provenances used in this research and the highest induction rate was 78.8% ± 12.5% (F2), the lowest was 62.50 ± 12.8% (F1). Since the protocol of ET induction was not modified significantly compared with the previously one^1^, the much higher induction rates in this research may result from the different explant sources and/or the better operation in details.

It could be mentioned, the proliferation of ET is not always in accordance with SE maturation. When the given parameters were evaluated with the four individual cell lines, the differences among them are apparent. It is a general knowledge that the desired protocol of somatic embryogenesis should fit all the cell lines. However, no such protocol could be developed in practice. In fact, a protocol that fits most cell lines will be selected in order to reduce the cost. This research revealed the remarked difference existed among the cultures that fit individual lines respectively. Thus, for elite cell lines, for example lines of high genetic gain, protocol development on the basis of genotype responses is strongly recommended if the general culture protocol does not function properly for specific cell lines that are essential in practice. On the results of this research, the general culture medium for ET proliferation for all the cell lines was mLV as the basal medium supplemented with 2.0 mg/L 2,4-D, 2 mg/L 6-BA, 10 g/L sucrose, 0.8 g/L casein hydrolysate, 0.5 g/L l-glutamine, and 4 g/L Gelrite, whereas on Sun and Jia^4^, 1/2LM medium was used as basal medium supplemented with 1.0 mg/L 2,4-D, 0.5 mg/L 6-B A, 0.5 mg/L KT and the highest embryonic callus proliferation rate was 102.27%.On Tao et al.^1^, it was 1/2LM supplemented with 6.25 µM (1.38 mg/L) 2,4-D, 3.75 µM (0.84 mg/L) BA for liquid cultures and 7.5 µM (1.69 mg/L) BA, 12.5 µM (2.76 mg/L) 2,4-D for solid cultures, and the highest proliferation rate of embryonic callus was 179.1%. With the optimized protocol in this study, the ET proliferation rate could increase up to 1473.7 ± 556.0%. In addition, in previous studies, the culture conditions of ET proliferation, and SE maturation were often considered separately. Few studies were reported on the influence of the cultures, i.e. the previous culture stage on the following one. This study found that the culture components in the ET proliferation stage not only affected ET proliferation, and also impacted SE maturation at the following stage by the specific concentrations of 2,4-D and 6-BA, which has not been reported in previous studies in blue spruce.

## Data Availability

Data and materials of the current study are available from the corresponding author upon reasonable request. All data generated and/or analyzed during this study are included in this published article.
